# Characterization of Satellite DNAs in Squirrel Monkeys genus *Saimiri* (Cebidae, Platyrrhini)

**DOI:** 10.1038/s41598-020-64620-1

**Published:** 2020-05-08

**Authors:** Mirela Pelizaro Valeri, Guilherme Borges Dias, Camila Nascimento Moreira, Yatiyo Yonenaga-Yassuda, Roscoe Stanyon, Gustavo Campos e Silva Kuhn, Marta Svartman

**Affiliations:** 1 Laboratório de Citogenômica Evolutiva, Departamento de Genética, Ecologia e Evolução, Instituto de Ciências Biológicas, Universidade Federal de Minas Gerais, Belo Horizonte, MG Brazil; 2 Department of Genetics and Institute of Bioinformatics, University of Georgia, Athens, GA United States of America; 3 Departamento de Genética e Biologia Evolutiva, Instituto de Biociências, Universidade de São Paulo, São Paulo, SP Brazil; 4 Department of Biology, University of Florence, Florence, Italy

**Keywords:** Molecular evolution, Phylogenetics, Taxonomy, Cytogenetics, Evolutionary biology, Genome

## Abstract

The genus *Saimiri* is a decades-long taxonomic and phylogenetic puzzle to which cytogenetics has contributed crucial data. All *Saimiri* species apparently have a diploid number of 2n = 44 but vary in the number of chromosome arms. Repetitive sequences such as satellite DNAs are potentially informative cytogenetic markers because they display high evolutionary rates. Our goal is to increase the pertinent karyological data by more fully characterizing satellite DNA sequences in the *Saimiri* genus. We were able to identify two abundant satellite DNAs, alpha (~340 bp) and CapA (~1,500 bp), from short-read clustering of sequencing datasets from *S. boliviensis*. The alpha sequences comprise about 1% and the CapA 2.2% of the *S. boliviensis* genome. We also mapped both satellite DNAs in *S. boliviensis, S. sciureus, S. vanzolinii*, and *S. ustus*. The alpha has high interspecific repeat homogeneity and was mapped to the centromeres of all analyzed species. CapA is associated with non-pericentromeric heterochromatin and its distribution varies among *Saimiri* species. We conclude that CapA genomic distribution and its pervasiveness across Platyrrhini makes it an attractive cytogenetic marker for *Saimiri* and other New World monkeys.

## Introduction

Squirrel monkeys of the genus *Saimiri* (Cebidae, Platyrrhini) are medium sized neotropical primates inhabiting forest environments of South America. They range from about 10°N to 17°S including the Amazon basin, the Guianas, and coastal zones of Central America^1,2^. As for many other New World monkey (NWM) taxa the phylogenetic relationships within the genus *Saimiri* are still debated^3^. Even the number of species is uncertain, historically ranging from one to 16 distinguished species^4–11^. In a recent molecular report on mitochondrial D-Loop and cyt *b* sequences, Alfaro *et al*.^3^ presented a provisional taxonomy of seven *Saimiri* species and various subspecies: (1) *S. boliviensis*, (2) *S. cassiquiarensis* (*S*. *c*. *cassiquiarensis*, *S*. *c*. *albigena*, *S*. *c*. *macrodon* A, *S*. *c*. *macrodon* B, and *S*. *c*. *macrodon* C), (3) *S*. *collinsi*, (4) *S*. *oerstedii* (*S. o. oerstedii* and *S. o. citronellus*), (5) S*. sciureus*, (6) *S. ustus* (A, B, and C lineages), and (7) *S. vanzolinii*.

Cytogenetic studies consistently showed that all *Saimiri* have a diploid number of 2n = 44, but can differ in fundamental numbers (FN, the number of chromosome arms) which range from 74 to 78^12^. Differences in FN in *Saimiri* have traditionally been expressed as the number of acrocentric chromosomes, with five (FN = 78) to seven pairs (FN = 74). FN variation was thought to correlate with geographic distribution and taxonomy. According to Jones *et al*.^13^ individuals from Costa Rica, Panama and Iquitos – Peru had five acrocentric pairs (FN = 78), those originating from Leticia – Colombia had six pairs (FN = 76) and specimens from Georgetown – Guiana had seven pairs of acrocentric chromosomes (FN = 74). Differences in FN in *Saimiri* were previously thought to be the result of pericentric inversions and reciprocal translocations^14–16^ but recently Chiatante *et al*.^12^, using high-resolution BAC-FISH analysis, showed that centromere repositioning explains differences in FN in *Saimiri*. These authors, however, did not address the taxonomic issue within the genus.

A class of markers that can be used to study karyotype evolution and address taxonomic issues is satellite DNAs (satDNAs). These sequences consist of tandem repeats organized in large arrays (up to Mb size) typically associated with chromosome landmarks such as centromeres, telomeres, and heterochromatic regions [e.g.^17–19^, reviewed in^20^]. Several satDNAs show a concerted mode of evolution, in which new mutations are homogenized within satDNA arrays in a genome and differentially fixed in reproductively isolated populations [reviewed in^20–22^]. Mechanisms such as gene conversion and unequal crossing-over are involved in the evolutionary process known as molecular drive, responsible for the concerted evolution of satDNAs^21^. The rapid concerted evolution of satDNAs can result in high intraspecific sequence homogeneity and interspecific differences, making satDNAs potential taxonomic markers and, in some cases, allowing their use for phylogenetic inferences^20,23^. SatDNAs have been used as cytogenetic markers facilitating species identification in many taxa, including primates^24^, frogs^25^, fish^26^ and plants^27^.

The alpha is the most studied satDNA in primates. It has a centromeric location and its monomer length in Old World primates is ~170 bp. Most NWMs (Platyrrhini) have a derived alpha with ~340 bp but species of the Pitheciidae family have a monomer of ~550 bp composed by four ~170 bp subunits with the third one incomplete^17,28,29^. Alpha satDNA is often highly divergent among species and also among chromosomes of the same species^18,28^. CapA is a satDNA present only in NWMs, with ~1,500 bp monomer length and was found in the three Platyrrhini families, with different chromosome localization and abundance varying from less than 1% up to 5%^17,30,31^.

The repetitive DNA fraction of the *Saimiri* genomes, including their satDNAs, are largely unexplored. In this work, we employed bioinformatic and cytogenetic tools to characterize the satDNAs of *Saimiri*. We characterized the two most abundant satDNAs of the genus and used these sequences to analyze the karyotypes of several individuals. Alpha and CapA comprise ~1% and 2.2% of the *S. boliviensis* genome, respectively. The alpha satDNA has ~340 bp, a centromeric location and high interspecific monomer homogeneity, while CapA has ~1500 bp and is associated with constitutive heterochromatin. This satDNA was mainly located in distal regions of the short arms and in the interstitial heterochromatin of some chromosomes, showing different chromosome localization among *Saimiri* species. Novel markers may help to clarify the taxonomic and phylogenetic relationships among *Saimiri* taxa.

## Results

### Chromosome banding

The 12 *Saimiri* individuals analyzed presented the expected diploid number of 2n = 44, but their fundamental numbers (FNs) varied due to the presence of different numbers of acrocentric chromosomes, which ranged from 10 to 14 (five to seven pairs) (Table 1). The karyotypes were arranged according to Stanyon *et al*.^32^. GTG-banding allowed the identification of all chromosomes.

**Table 1:** Identification, sex, collection site, fundamental number and morphology of chromosome pairs 5 and 15 of the analyzed specimens.

Species	Specimen	Sex	Origin	FN	Pair 5	Pair 15
*S. boliviensis*	SBO1	Male	Unknown	76	SM	A
*S. sciureus*	SSC 782	Male	Presidente Figueiredo – Amazonas	74	A	A
*S. sciureus*	SSC 770	Female	Santarém – Pará	74	A	A
*S. sciureus*	SSC2	Male	Unknown	74	A	A
*S. vanzolinii*	SVA 321	Female	Lake Mamirauá, Tefé - Amazonas	76	SM	A
*S. vanzolinii*	SVA 322	Male	Lake Mamirauá, Tefé - Amazonas	76	SM	A
*S. ustus*	SUS 739	Male	Hydroelectric plant, Samuel - Rondônia	78	SM	SM
*S. ustus*	SUS 740	Female	Hydroelectric plant, Samuel - Rondônia	78	SM	SM
*S. ustus*	SUS 742	Female	Hydroelectric plant, Samuel - Rondônia	78	SM	SM
*S. ustus*	SUS 746	Female	Hydroelectric plant, Samuel - Rondônia	78	SM	SM
*S. ustus*	SUS 747	Female	Hydroelectric plant, Samuel - Rondônia	78	SM	SM
*S. ustus*	SUS786	Female	Hydroelectric plant, Samuel - Rondônia	78	SM	SM

SBO - *Saimiri boliviensis*; SSC - *Saimiri sciureus*; SVA - *Saimiri vanzolinii*; SUS - *Saimiri ustus*; FN – fundamental number; SM - submetacentric; A - acrocentric.

**Figure 1:**
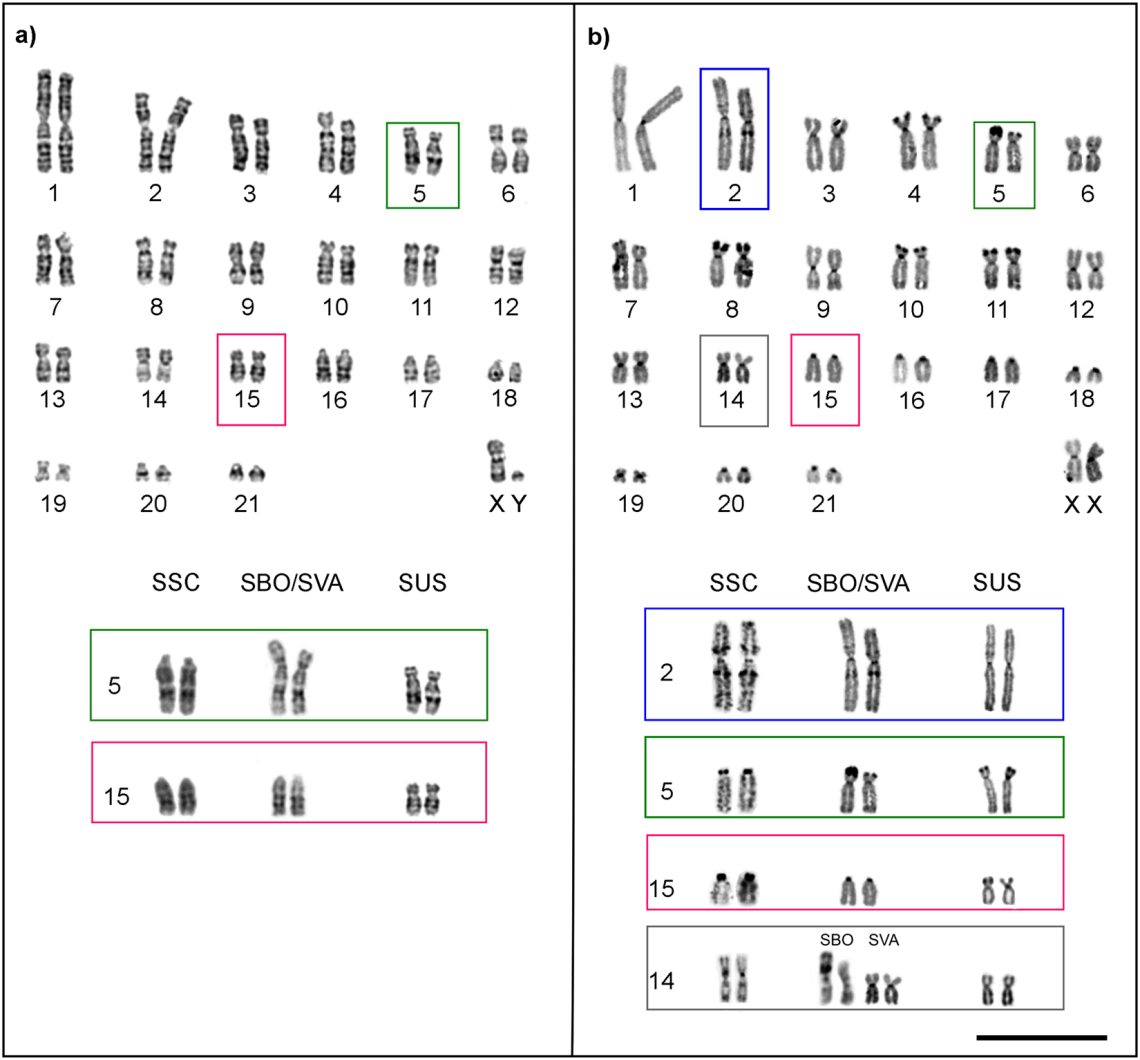
(**a**) Representative *Saimiri* karyotype with FN = 78 and five acrocentric pairs, pairs 5 and 15 are submetacentric (SUS 739); the lower panels show the acrocentric pairs 5 and 15 (SSC 770, SSC 782, SSC2); submetacentric pair 5 and acrocentric 15 (SBO1, SVA 321 and SVA 322), submetacentric pairs 5 and 15 (SUS). (**b**) CBG-banded *Saimiri* karyotype with FN = 76 (SVA 321), and in the boxes below pairs 2, 5, 15 and 14 from *S. sciureus* (SSC), *S. boliviensis* (SBO), *S. vanzolinii* (SVA) and *S. ustus* (SUS). Chromosome pair 2 is highlighted in blue, pair 5 in green, pair 14 in gray and pair 15 in pink. Bar = 10 μm.

The specimens identified as *S. sciureus* (SSC782, SSC770 and SSC2) had a FN = 74 and both pairs 5 and 15 were acrocentric. The *S. boliviensis* (SBO1) and the two *S. vanzolinii* specimens (SVA321 and SVA 322) had a FN = 76 with a submetacentric pair 5 and an acrocentric pair 15. All the *S. ustus* samples (SUS739, SUS740, SUS742, SUS746, SUS747 and SUS786) had a FN = 78 and both pairs 5 and 15 were submetacentric. These results supported previous conclusions^12^ that centromere shifts in pairs 5 and 15 explained the morphological variation of these chromosomes and the consequent differences in FNs (Fig. 1a; Supplementary Fig. S1).

CBG-banding revealed, in addition to the pericentromeric constitutive heterochromatin, the presence of distal and interstitial heterochromatic blocks in several chromosome pairs (Fig. 1b; Supplementary Fig. S2). The distribution and abundance of distal and interstitial heterochromatin was slightly different between species. Generally, distal CBG bands were located in the short arms of submetacentric chromosomes. In all analyzed specimens, heterochromatic blocks were detected in the distal regions of the short arms of pairs 4, 7, 8, 10, 11, and 13 and in the proximal regions of both arms of chromosome 2. Interstitial CBG bands in both arms of chromosome 2 were more evident in *S. sciureus* than in *S. vanzolinii* and were very light in *S. boliviensis* and all *S. ustus*. Pairs 5 and 15 had distal CBG bands in their short arms only in their submetacentric form. The *S. sciureus* specimens SSC770, SSC782 and SSC2 had the acrocentric form of chromosomes 5 and 15 and thus did not present the distal CBG bands in these pairs. The *S. boliviensis* and *S. vanzolinii* specimens showed distal CBG bands in pair 5. Distal CBG bands were detected in pairs 5 and 15 of all *S. ustus* analyzed. In SSC782 and SBO1, a CBG band was detected only in one homologue of pair 14, in the proximal and distal regions of the short arm.

### Satellite DNAs identification and chromosome mapping

After careful analysis of RepeatExplorer’s results, we identified two clusters that corresponded to potential satDNAs. Cluster 5 (CL5) comprises 22,193 reads (out of 2,230,692), representing ~1% of the *S. boliviensis* genome. RepeatExplorer includes partial assembly of reads into contigs and RepeatMasking of contigs using the RepBase metazoan library. This analysis indicated that CL5 corresponded to the centromeric repeat. Extending the search for CL5 sequences to the nr/nt GenBank database revealed that this cluster represents the well-known alpha satDNA, known to have a centromeric location in simian primates. Similarity searches of CL5 sequences against the *S. boliviensis* reference genome (accession GCA_000235385.1) revealed that these sequences are organized in tandem and that monomers are ~340 bp in length, which was confirmed by PCR in all *Saimiri* analyzed species (Supplementary Fig. S3a).

**Figure 2:**
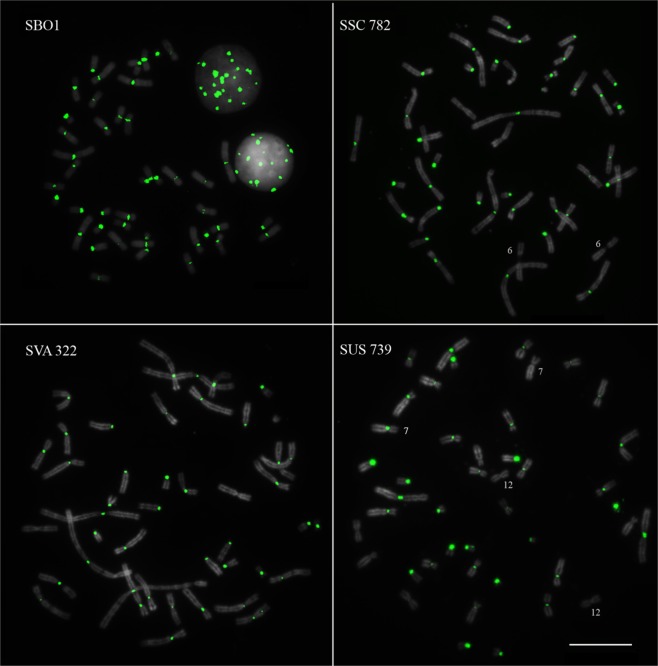
Metaphases of *S*. *boliviensis* (SBO1), *S*. *sciureus* (SSC 782), *S*. *vanzolinii* (SVA 322) and *S*. *ustus* (SUS 739) after FISH with the alpha satDNA probe. The signal in pair 6 of SSC 782 was weaker than in the other species; pair 12 and one homologue of pair 7 of SUS 739 did not show any signal. Bar = 10 μm.

Fluorescent *in situ* hybridization (FISH) with the alpha satDNA in squirrel monkey chromosomes revealed its presence in the centromeric region of all chromosomes in *S. boliviensis*, *S. sciureus*, and *S. vanzolinii* (Fig. 2). In *S. ustus* it was absent from pair 12 and from one homologue of pair 7. Furthermore, the hybridization signal in chromosome 6 of SSC770, SSC 782 and SSC2 was much weaker when compared to the other species. In all *Saimiri* species, the alpha satDNA signal was more intense in acrocentric chromosomes and in the metacentric pair 19.

The second potential satDNA cluster, CL3, was initially found split into four clusters with a strongly connected component to each other (CL6, CL7, CL8 and CL12). These were merged using the RepeatExplorer cluster merger tool, thus recovering a larger CL3. This cluster comprises 49,193 reads, or ~2.2% of the genome, representing the second most abundant repeat family in the *S. boliviensis* genome (Supplementary Table S1). Analysis of this sequence in the *S. boliviensis* reference genome revealed tandem repeats with a ~1,500 bp monomer length confirmed by PCR (Supplementary Fig. S3b). Similarity searches on the nr/nt database from GenBank using partially assembled contigs from the reads of CL3 as queries revealed that this sequence is homologous to a satDNA named CapA described in *Sapajus apella*^17^.

**Figure 3:**
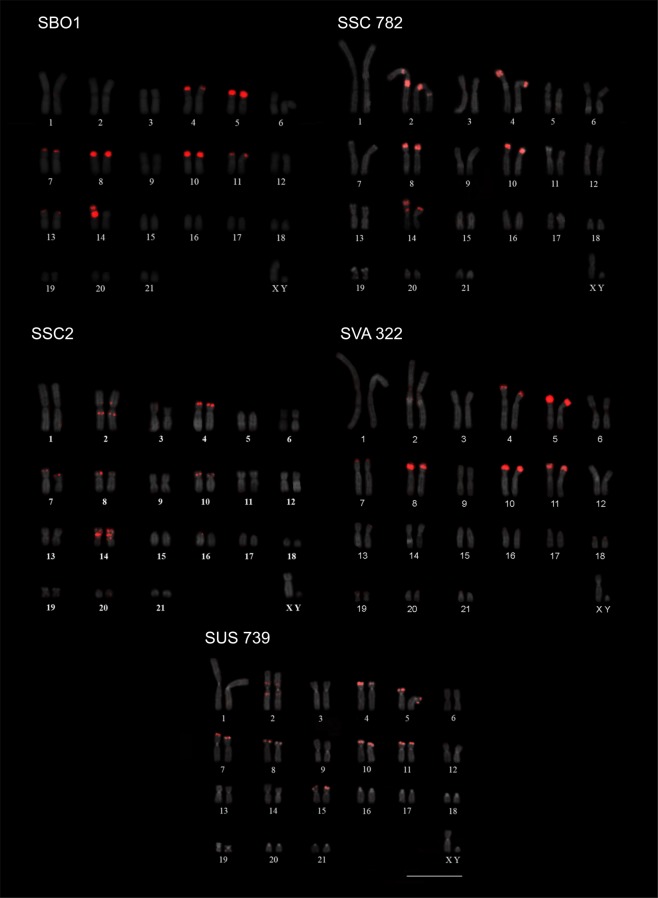
FISH with CapA in the karyotypes of *S. boliviensis* (SBO1), *S. sciureus* (SSC 782 and SSC2), *S. vanzolinii* (SVA 322) and *S. ustus* (SUS 739). Bar = 10 μm.

We have previously obtained a probe for CapA when studying the origin and distribution of this satDNA in mammals^31^ and we used the same probe to analyze CapA distribution in the *Saimiri* genus. FISH with the CapA probe showed a distribution largely coincident with the heterochromatic regions revealed after CBG-banding in the four *Saimiri* species. Signals were mainly located in the distal regions of the short arms of submetacentric chromosomes and in the interstitial heterochromatin of some chromosome pairs (Fig. 3). There was a slight variation in CapA localization among *Saimiri* species. The difference of CapA localization between *S. boliviensis* and *S. vanzolinii*, that share the same FN = 76, was its presence in chromosome 14 of *S. boliviensis* and its absence in the same chromosome of *S. vanzolinii*. Besides *S. boliviensis*, only *S. sciureus* had CapA mapped to pair 14. Only in *S. sciureus* (FN = 74) CapA was not detected in pair 11 and its signal was much more intense in pair 2. CapA was not detected in pair 13 of *S. sciureus* and *S. ustus* (FN = 78). As observed with CBG bands, pairs 5 and 15 had distal CapA signals in their short arms only in their submetacentric form. Thus, *S. sciureus* did not show CapA in pairs 5 and 15; *S. boliviensis* and *S. vanzolinii* had the satDNA mapped in pair 5 and *S. ustus* in both pairs.

### Alpha satDNA sequence analysis

**Figure 4:**
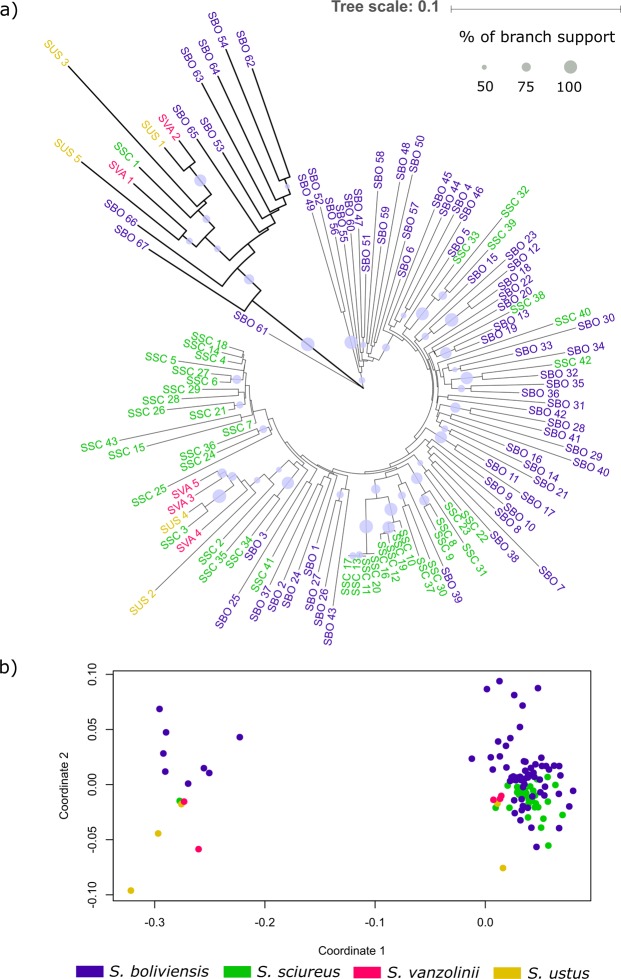
(**a**) Neighbor-joining clustering of alpha sequences of *S. boliviensis* (SBO), *S. sciureus* (SSC), *S. vanzolinii* (SVA) and *S. ustus* (SUS) inferred by the neighbor-joining method with 1000 bootstrap replicates. Tree visualized in iTOL v4.3.3 (https://itol.embl.de/)^52^. (**b**) Non-metric multidimensional scaling (NMDS) of evolutionary divergence among the same alpha sequences used in the phylogenetic analyses. The scaling represents euclidean distances for two dimensions (stress: 0.1127148). The sequences present in the left group of ordinations are the same of the branch in bold of the phylogenetic tree. Colors indicate the sequence taxa (bottom).

Sequences of the alpha satDNA were obtained for all four *Saimiri* species studied herein from either the reference genome (*S. boliviensis*), from clones obtained by Kugou *et al*.^33^ for *S. sciureus*, and from cloning and Sanger sequencing performed in this study (*S. sciureus*, *S. vanzolinii* and *S. ustus*). These sequences were aligned and compared for intra and interspecies diversity, using neighbor-joining clustering and non-metric multidimensional scaling (NMDS) ordinations. Alpha sequences showed the same value (0.16 substitutions per site) for intra and interspecific diversity. Both the neighbor-joining tree and NMDS ordinations revealed that alpha sequences do not display species-specific clustering (Fig. 4). Neighbor-joining clustering suggested a divergent group of alpha sequences, but in a branch without support. Interestingly, NMDS showed the same group of alpha sequences clearly distinct from the others, indicating a potential chromosome variant. The neighbor-joining and NMDS results were supported by the Maximum Likelihood method (Tamura 3-parameter model) analysis (Supplementary Fig. S4).

## Discussion

We identified two abundant satDNAs in the genome of *Saimiri boliviensis*, alpha and CapA. We characterized both for repeat size, abundance, and chromosome localization in *S. boliviensis*, *S. sciureus S. vanzolinii*, and *S. ustus*. The alpha satDNA comprises ~1% of the *S. boliviensis* genome and its ~340 bp monomer length was confirmed in all four *Saimiri* species. This repeat structure with ~340 bp is found in most NWMs and is probably the ancestral form in the group, evolved through a duplication of the ~170 bp monomer found in Catarrhini^18,29^.

**Figure 5:**
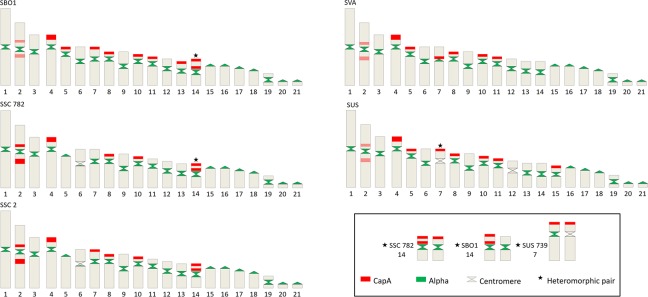
Schemes of *Saimiri* chromosomes showing the localization of the alpha and CapA satDNAs in *S. boliviensis* (SBO), *S. sciureus* (SSC 782 and SSC2), *S. vanzolinii* (SVA) and *S. ustus* (SUS). The heteromorphic pairs for satDNAs distribution are depicted in the inset.

The alpha satDNA centromeric localization was observed in all chromosomes of *S. boliviensis*, *S. sciureus* and *S. vanzolinii*. Moreover, it appears to be absent or undetectable by FISH (due to low repeat number or sequence divergence) from chromosomes 7 and 12 in *S. ustus* (Fig. 5). Variable FNs among squirrel monkey species is due to centromere repositioning in pairs 5 and 15^12^. Our sample includes specimens with both morphologies of chromosomes 5 and 15 and the alpha satDNA was always detected at their centromeres, indicating that these evolutionary new centromeres (ENCs) are mature.

The alphoid satDNA family is known to be part of the centromere in simian primates (Catarrhini and Platyrrhini). In a phylogenetic analysis of primate alpha satDNA, Alkan *et al*.^18^ reported that alpha satDNA repeats are evolutionarily distinct and heterogenous between human, chimpanzee and macaques. Alpha DNA was shown to have a genus-specific chromosome localization in small apes from the *Nomascus* and *Hylobates* genera, which could be used as a cytogenetic marker^24^.

Our sequence analysis of alpha repeats between *Saimiri* species revealed a high interspecific repeat homogeneity. The homogeneity found among alpha repeats of all analyzed species may be due to the recent *Saimiri* species diversification (1.4–0.8 million years ago-Mya)^3^ and/or to hybridization events. In fact, hybridization has been reported between *S. sciureus* and *S. ustus*^34^, between *S. boliviensis* and *S. sciureus*, and between *S. boliviensis* and *S. ustus*^35^. Another possibility is that the alpha repeats have been conserved due to their important role in centromere function and maintenance. For example, Smalec *et al*.^36^ reported a conserved centromeric repeat (PM sat) among rodents of the genus *Peromyscus* and suggested that molecular drive was not the only agent in the evolution of this satDNA and that the homologous arrays may play a role in the chromosome evolution of the genus.

NMDS ordinations and neighbor-joining clustering clearly split *Saimiri* alpha repeats into two groups, one containing most sequences and the other containing only a few sequences but representing all four species. This could be an indication that monomers are evolving divergently among the chromosomes. In humans, alpha is known to have chromosome-specific sequences^37^, and in *Callitrix jacchus* alpha repeats are divided into seven clusters that showed different hybridization patterns among chromosomes^29^.

The second satDNA analyzed, CapA, has ~1,500 bp monomers, comprises ~2.2% of the *S. boliviensis* genome and is associated with constitutive heterochromatin. It is the second most abundant repetition in *S. boliviensis*, only surpassed by Alu transposable elements (Short Interspersed Nuclear Elements – SINEs). CapA was first identified after digestion of *Sapajus apella* (previously classified as *Cebus apella*) genomic DNA with restriction enzymes, and was shown to be ~1,500 bp long and to comprise about 5% of the genome^17,30^. In a recent study, we provided evidence that the CapA satDNA is homologous to an intronic sequence of the NOS1AP gene (*Homo sapiens nitric oxide synthase 1 adaptor protein*), and likely originated from this single-copy sequence through duplication and unequal crossing-over^31^. We also showed that CapA is present in representatives of the three Platyrrhini families (Cebidae, Atelidae and Pitheciidae; except in Callitrichines and in the *Callicebus* genus) with different genome abundance and chromosome localization, always associated with constitutive heterochromatin.

CapA repeats in *Saimiri* chromosomes are mostly enriched in the distal heterochromatin of submetacentric chromosomes, but the overall distribution and abundance differed among species (Fig. 5).

Interestingly, CapA was mapped in some chromosomes involved in rearrangements. For instance, CapA distribution in *Saimiri* chromosomes 5 and 15 was coincident with the CBG bands: they were absent in the acrocentric forms and present in the distal heterochromatin of the short arm in the submetacentric variants of these chromosomes. This variation may be related to the morphology of these chromosomes and perhaps with the process of ENCs formation in *Saimiri*. According to the putative ancestral Platyrrhini karyotype, the ancestral morphology of chromosome 5 and 15 is submetacentric and acrocentric, respectively^32,38^. Taking this into account, chromosome 5 would have lost CapA sequences when the chromosome became acrocentric and the new centromere occupied this region. Conversely, in chromosome 15, the CapA satDNA would have colonized the old centromere region after the chromosome became submetacentric.

SBO1 was previously analyzed by Chiatante *et al*.^12^, who reported pair 14 as heteromorphic, with isoforms A and B differing by a paracentric inversion and two heterochromatic blocks, one distal and one in the proximal region of the short arm. This same polymorphism was also described in several specimens of squirrel monkeys, especially in those from Bolivia, in which heterozygotes for pair 14 were more common than homozygotes^14,39^. Chiatante *et al*.^12^ suggested that an inversion in the isoform B may have carried some telomeric DNA sequences to the interstitial region of the short arm, explaining the heterochromatic blocks. Three of our specimens (SBO1, SSC 782 and SSC2) presented the isoform B of chromosome 14, and in all of them CapA was located in the interstitial heterochromatin and distal region. In SSC 782, CapA was also detected in the distal region of the short arm of isoform A. This indicates that CapA could have been involved in the generation of this paracentric inversion, instead of the suggested telomeric sequences^12^.

Chromosome painting with human probes revealed that *Saimiri* 2q is formed by homologues of human chromosomes 14 and 15 (HSA 14 and 15) and their signals are intercalated due to inversions^32^. CapA was mapped on 2q in a region that may correspond to the breakpoint of one of this inversions. CapA was also observed in *Saimiri* 2p, which is homologous to HSA 9^32^. In *Sapajus apella* HSA 9 corresponds to pair 19, which has an interstitial heterochromatic block where CapA was observed and corresponds to an inversion^17,40^. Pair 7 of *Saimiri* is formed by the association of HSA 15/HSA 2^32^ and presents CapA in the short arm of most specimens analyzed (Fig. 5), although seemingly not at the association region.

Although our data do no allow to pinpoint CapA to chromosome rearrangements, they indicate that a detailed analysis of these regions could provide some new interesting data.

CapA distribution differed more among *Saimiri* species than CBG bands. Even individuals of different species with the same karyotype and FN differed in their CapA localization, as for instance, *S. boliviensis* and *S. vanzolinii*, both with FN = 76 (Fig. 3). The specimens identified as *S. sciureus* (FN = 74) did not show CapA in pair 11 and the satDNA was more abundant in pair 2 when compared with the other species (Fig. 3). The specimen SSC 770 identified as *S. sciureus* has a similar karyotype and CapA distribution to SSC 782, however its collection site suggests that it may actually be a *S. collinsi* according to Alfaro *et al*.^3^. No karyotype has been described for *S. collinsi* or for samples collected within its geographic distribution, preventing us from further conclusions.

The variable chromosomal localization of CapA among the *Saimiri* species analyzed herein suggests that, combined with FN, this sequence may be used as a valuable tool in taxonomic identification. Hybridization in squirrel monkeys has been reported in captivity and in nature^4,6,35^ and CapA mapping may also reveal the origin of chromosome sets in hybrids more precisely than chromosome morphology or banding patterns.

The rapid expansion and diversification of squirrel monkeys occurred in the Amazon basin with all the speciation events estimated in the range between 1.4–0.8 Mya^3^ starting with the divergence between *S. boliviensis* and the ancestor to all other squirrel monkeys at 1.4–1.6 Mya^2^. Despite the very short time since diversification, we were able to detect different CapA chromosomal distributions among the *Saimiri* specimens analyzed. Conversely, the alpha satDNA displayed high interspecific repeat homogeneity which could be related to its role in centromere function and maintenance.

The cytogenetic information about *Saimiri* is poor when compared to morphological and molecular data. The number of specimens karyotypically analyzed is low, and most samples are not geotagged. Until now, the karyotypes of only five out of the seven recently recognized species^3^ have been reported and they are very similar, including their banding patterns. In order to further validate CapA as a marker, more geotagged specimens need to be analyzed. The study of species not yet karyotyped will also help clarify *Saimiri*’s taxonomic puzzle and inform its systematics.

Our results indicate that CapA is a promising cytogenetic marker and that it could be useful for taxonomic, phylogenetic and conservation studies of *Saimiri*. In addition, the fact that CapA is present across Platyrrhini further extends its utility as a marker for chromosome and genome evolution studies in NWMs^31^. The availability of new markers is especially important in the face of threats of extinction to an alarming large number of NWM species due to rapid habitat loss^41^.

## Materials and Methods

### Biological samples and chromosome banding

Chromosome spreads and genomic DNAs were obtained from fibroblast cultures of 12 squirrel monkeys: one *S. boliviensis*, three *S. sciureus*, two *S. vanzolinii*, and six *S. ustus*. We used previously established cell lines and the available details for the samples are provided in Table 1. The work did not involve the direct use of animals, so ethical permission was not required. Genomic DNAs were purified with the Wizard Genomic DNA Purification Kit (Promega) and chromosome spreads were obtained from cultured cells according to Stanyon and Galleni^42^. GTG- and CBG-banding were performed according to Seabright^43^ and Sumner^44^, respectively. The samples from *S. boliviensis* (SBO1) were previously analyzed in Chiatante *et al*.^12^ and Capozzi *et al*.^45^. The *S. sciureus* (SSC2) sample was previously reported in Chiatante *et al*.^12^. The two *S. vanzolinii* (SVA 321 and SVA 322) samples were previously reported in Yonenaga-Yassuda and Chu^46^.

### Satellite DNAs identification

The RepeatExplorer pipeline^47,48^ was used to identify satDNAs based on all to all similarity comparison of the Illumina reads of *S. boliviensis* (NCBI SRA access: SRR317821). A total of 2,230,692 Illumina reads (~100 bp long) comprising 7% of the estimated *S. boliviensis* genome were randomly sampled and used in this analysis. The results are represented as graph-based clusters of similar reads and the shape of the clusters is indicative of the nature of the different repeat families (e.g. globula and ring-like structures suggest tandemly organized repeats). The number of reads within the clusters (out of the total used in the analysis) indicates the abundance of that cluster in the genome. The clustering process tends to split large repeats into several clusters, but the pipeline has a separate re-clustering tool for a user-aided merging of the clusters. The reads that make up each cluster are partially assembled into contigs that can be used for repeat annotation. The minimum overlapping lengths used for clustering and assembly were 55 and 40 bp, respectively. Sequences in clusters with globular/ring-like structure were analyzed in detail through similarity searches against the *S. boliviensis* reference genome (accession GCA_000235385.1) using the BLASTn tool with default parameters^49^. Additionally, satDNA clusters were annotated through similarity searches against the whole non-redundant nucleotide collection in GenBank.

### PCR amplification, cloning and sequencing of satellite DNAs

Isolation of alpha satDNA was performed through polymerase chain reaction (PCR) of *Saimiri* genomic DNAs using the following specific primer set: alpha-F (ACAGGGAAATATCTGCTTCTAAATC) and alpha-R (GCTTACTGCTGTTTCTTCCATATG). The thermocycling conditions were as follows: 95 °C—3 min, 35 cycles: 95 °C—30 sec; 60 °C—30 sec; 72 °C—1 min; final elongation: 72 °C—3 min. The repeat monomers obtained by PCR were cloned into pGEM-T Easy vector plasmids (Promega) and used to transform *E*. *coli* strain XL1-BLUE (Phoneutria) through electroporation. Recombinant colonies were capillary sequenced with the ABI3130 platform (Applied Biosystems) and are available in GenBank under accession numbers MK879580-MK879592. We obtained three sequenced clones from *S. sciureus*, five from *S. vanzolinii* and five from *S. ustus*. These sequenced clones were used in molecular analyses and as FISH probes. CapA amplification was performed using genomic DNAs from *S. boliviensis* and *S. vanzolinii* and the primer set CapA-F (ACTTCCTCACTGACCTGTCTT) and CapA-R (GGGCTGATGCTTAATGTAGCA). CapA isolation and cloning were previously performed in Valeri *et al*.^31^ using human DNA and the same primer set described. The sequenced product is deposited in GenBank under the accession number MG264524 and was used as a FISH probe in this study.

### Fluorescent *in situ* hybridization (FISH)

FISH was performed using alpha and CapA sequences as probes on metaphase spreads of the four *Saimiri* species. SatDNA probes were prepared from pGEM-T Easy cloned sequences and labeled by nick translation with biotin-16-dUTP or digoxigenin-11-dUTP (Nick Translation mix, Roche Applied Science). Chromosomes were denatured in 70% formamide/2xSSC (saline-sodium citrate buffer) at 75% for 105 sec. The hybridization mix consisted of 100 ng of labeled probe in 50% formamide/2xSSC and was denatured for 10 min at 98 °C and added to the chromosome spreads. Hybridization was carried at 37 °C for 16–20 hours. Post-hybridization washes consisted of three baths of 2xSSC at 45 °C for 5 min each. Immunodetection was performed with neutravidin+rhodamine or avidin+FITC conjugates (Roche Applied Science) and the slides were mounted with DAPI 1:500 in Slowfade (Life Technologies). Chromosome identification was based on the Q-banding pattern produced after DAPI staining. The analyses were performed under a Zeiss Axioimager 2 epifluorescence microscope equipped with a CCD camera and image acquisition was performed with the AxioVision software (Carl Zeiss MicroImaging, Jena, Germany).

### Alpha satDNA sequence analyses

In order to test if satDNA sequences of a species showed signs of concerted evolution we aligned the satDNA sequence monomers with MUSCLE^50^ and performed clustering using the neighbor-joining method with 1000 bootstrap replicates in MEGA X^51^. Alpha sequences intra and interspecific diversities were calculated using the Maximum Composite Likelihood model in MEGA X. In addition, we computed pairwise distances between all sequences using a Maximum Composite Likelihood model in MEGA X and used these data to perform a NMDS analysis. The dataset used for these analyses was mainly composed of alpha monomeric sequences retrieved from GenBank (https://www.ncbi.nlm.nih.gov/genbank/). Thus, we performed BLASTn using the alpha consensus sequence as query against the *S. boliviensis* reference genome (accession GCA_000235385.1) and *S. sciureus* alpha clones (accession LC075928-LC075953) obtained by Kugou *et al*.^33^. Because GenBank lacks data from *S. vanzolinii* and *S. ustus*, we added sequences of these species obtained herein by cloning and Sanger sequencing. The total of alpha monomeric sequences used was 120, composed by 67 from *S. boliviensis*, 43 from *S. sciureus*, five from *S. vanzolinii* and five from *S. ustus*. The resulting neighbor-joining tree was visualized in iTOL v4.3.3 (https://itol.embl.de/)^52^. NMDS ordinations were generated with the R package vegan^53^, representing the divergence between sequences values in Euclidian distances for two dimensions. RStudio v1.1.463^54^ was used to conduct the NMDS analyses and to plot the ordinations.

## Supplementary information

Supplementary information.

## Data Availability

The datasets generated during and/or analyzed in the current study are available in the GenBank repository (https://www.ncbi.nlm.nih.gov/genbank/) and in NCBI trace and short-read archive (https://trace.ncbi.nlm.nih.gov/Traces/sra/sra.cgi?). All accession numbers are provided in the Methods section.
