# Publisher Correction: Characterization of Satellite DNAs in Squirrel Monkeys genus *Saimiri* (Cebidae, Platyrrhini)

**DOI:** 10.1038/s41598-020-70051-9

**Published:** 2020-08-06

**Authors:** Mirela Pelizaro Valeri, Guilherme Borges Dias, Camila Nascimento Moreira, Yatiyo Yonenaga-Yassuda, Roscoe Stanyon, Gustavo Campos e Silva Kuhn, Marta Svartman

**Affiliations:** 1 Laboratório de Citogenômica Evolutiva, Departamento de Genética, Ecologia e Evolução, Instituto de Ciências Biológicas, Universidade Federal de Minas Gerais, Belo Horizonte, MG Brazil; 2 Department of Genetics and Institute of Bioinformatics, University of Georgia, Athens, GA United States of America; 3 Departamento de Genética e Biologia Evolutiva, Instituto de Biociências, Universidade de São Paulo, São Paulo, SP Brazil; 4 Department of Biology, University of Florence, Florence, Italy

Correction to: *Scientific Reports*10.1038/s41598-020-64620-1, published online 8 May 2020

In the original version of this Article, the author Gustavo Campos e Silva Kuhn was incorrectly indexed. This error has now been corrected.

