# Supplementary material for: Characterization of Satellite DNAs in Squirrel Monkeys genus Saimiri (Cebidae, Platyrrhini)
Source: Sci Rep. 2020 May 8;10:7783. doi: 10.1038/s41598-020-64620-1 (PMC7210261; doi:10.1038/s41598-020-64620-1)
Supplement: Supplementary file 1 — Supplementary information. [file 41598_2020_64620_MOESM1_ESM.pdf]

Supplementary Information for

Characterization of Satellite DNAs in Squirrel Monkeys genus *Saimiri* (Cebidae, Platyrrhini)

Mirela Pelizaro Valeri[1]; Guilherme Borges Dias[2]; Camila Nascimento Moreira[3]; Yatiyo Yonenaga-Yassuda[3]; Roscoe Stanyon[4]; Gustavo Campos Silva Kuhn[1]; Marta Svartman[1*]

[1]Laboratório de Citogenômica Evolutiva, Departamento de Genética, Ecologia e Evolução, Instituto de Ciências Biológicas, Universidade Federal de Minas Gerais; Belo Horizonte, MG, Brazil.

[2]Department of Genetics and Institute of Bioinformatics, University of Georgia, Athens, GA, United States of America

[3]Departamento de Genética e Biologia Evolutiva, Instituto de Biociências, Universidade de São Paulo, SP, Brazil.

[4]Department of Biology, University of Florence, Florence, Italy

*Corresponding author
E-mail: svartmanm@ufmg.br

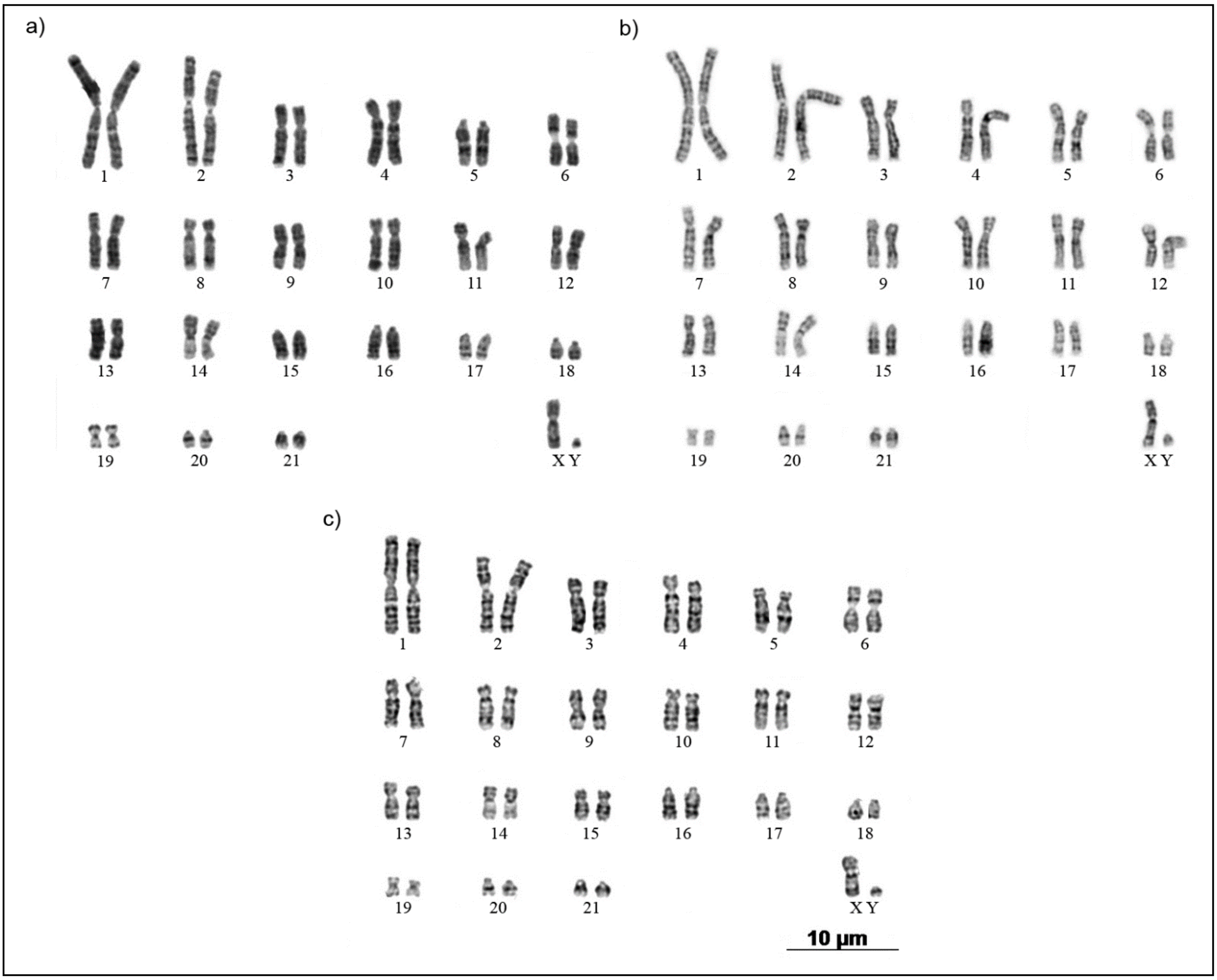

Supplementary Figure S1. Representative *Saimiri* karyotypes of (a) *S. sciureus* with five acrocentric pairs, chromosomes 5 and 15 are submetacentric (FN=78) from cell line SSC 782; (b) *S. boliviensis* and *S. vanzolinii* with six acrocentric pairs, chromosome 5 is submetacentric and 15 is acrocentric (FN=76) from cell line SVA 322; (c) *S. ustus* with seven acrocentric pairs, chromosomes 5 and 15 are acrocentric (FN=74) from cell line SUS 739.

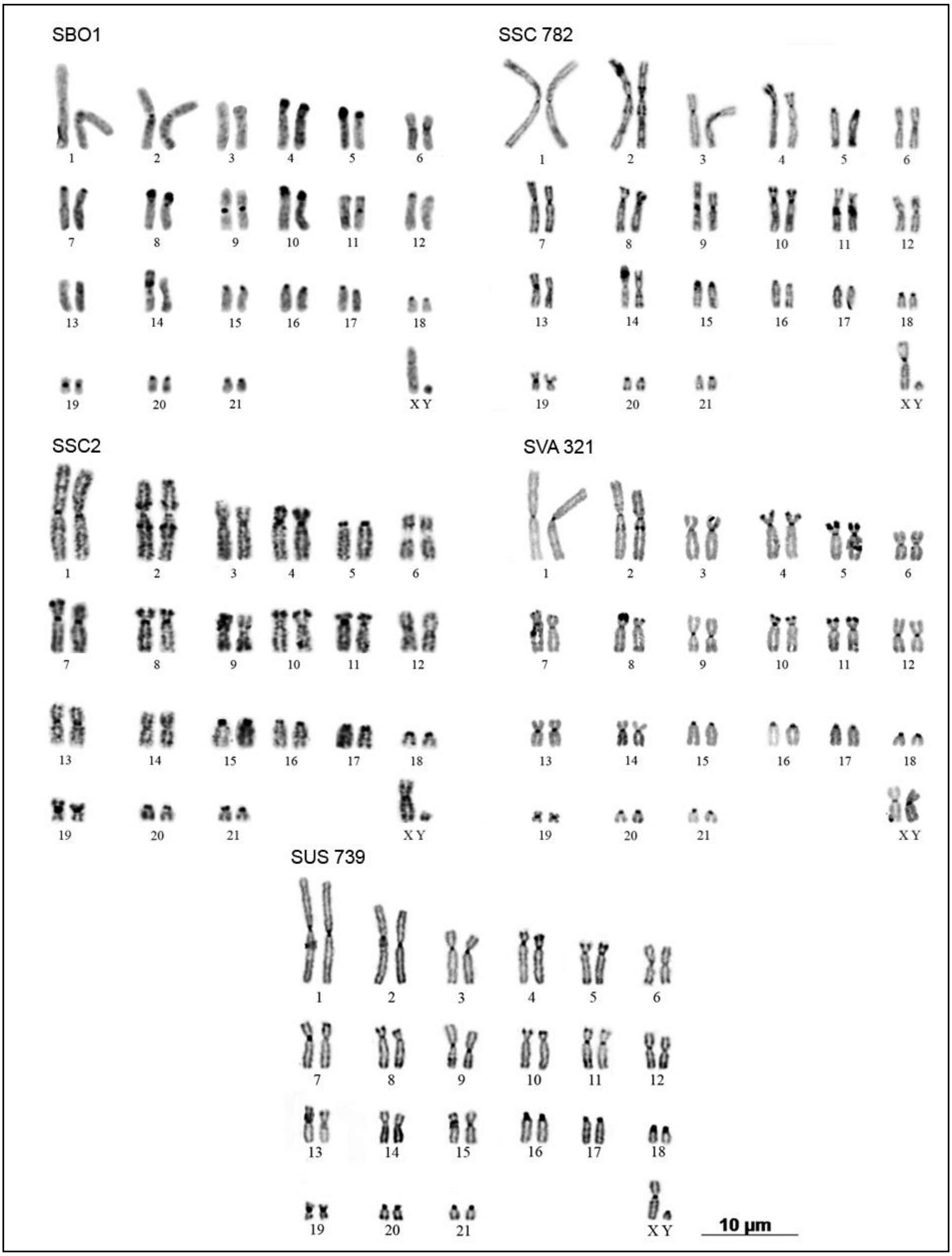

Supplementary Figure S2. CBG-banded *Saimiri* karyotypes of *S. boliviensis* (SBO1), *S. sciureus* (SSC 782 and SSC2), *S. vanzolinii* (SVA 322) and *S. ustus* (SUS 739).

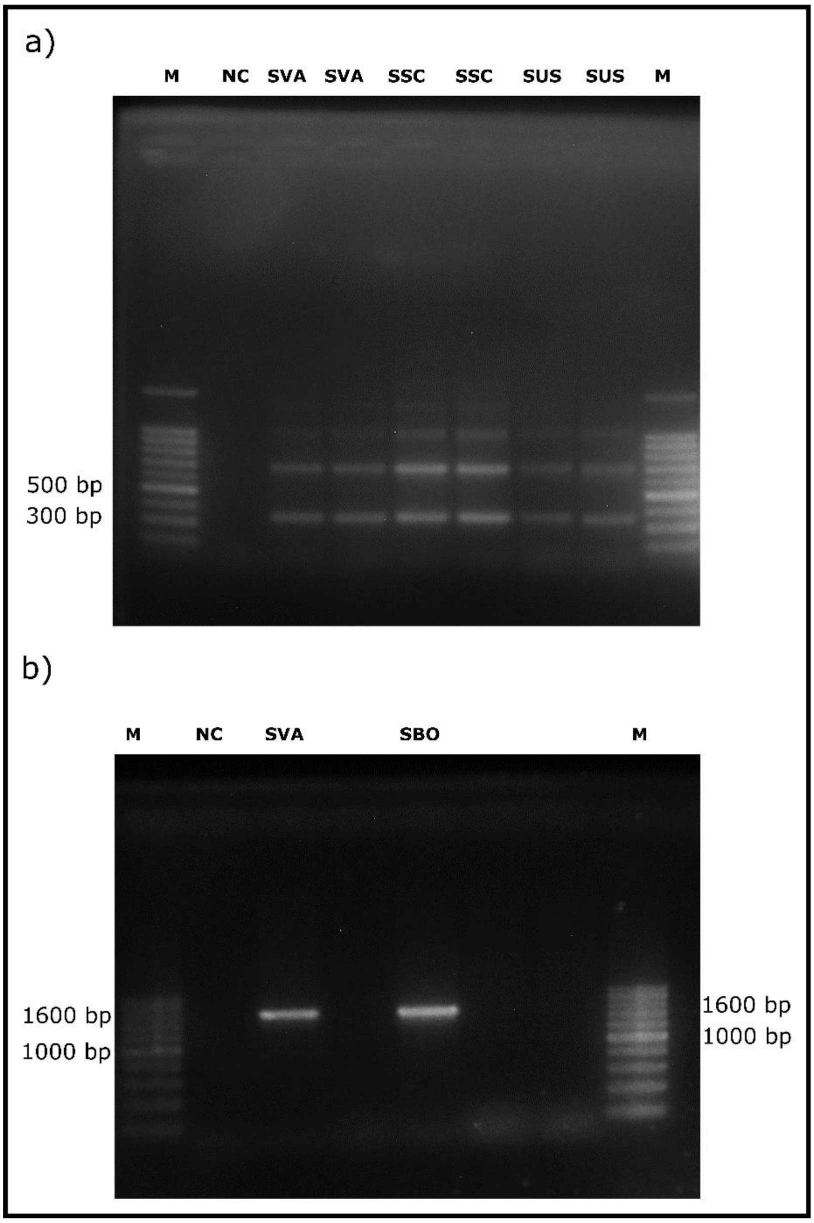

Supplementary Figure S3. Agarose gel (1%) showing the PCR products of *Saimiri* species using a) Alpha and b) CapA primers. M - DNA size marker (a) 100 bp ladder; b) 200 bp ladder); NC – Negative control (no DNA); SBO - *Saimiri boliviensis;* SSC - *Saimiri sciureus; SVA - Saimiri vanzolinii; SUS - Saimiri ustus.*

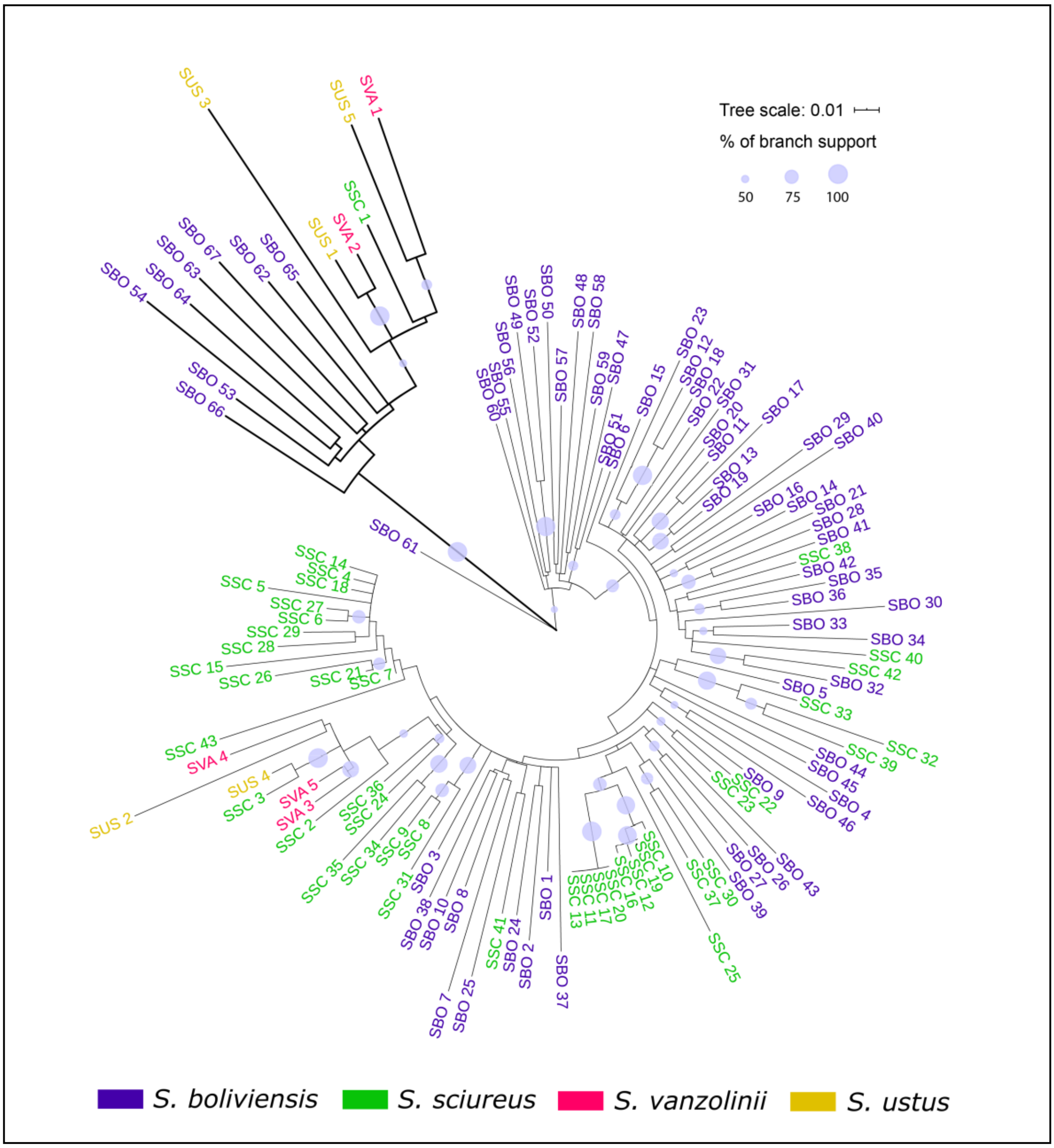

Supplementary Figure S4. Phylogenetic analysis of alpha sequences of *S. boliviensis* (SBO), *S. sciureus* (SSC), *S. vanzolinii* (SVA) and *S. ustus* (SUS) inferred by the Maximum Likelihood method (Tamura 3-parameter model) from 500 replicates using MEGA X. Tree visualized in iTOL v4.3.3 (https://itol.embl.de/).

Supplementary Table S1. Clusters retrieved by RepeatExplorer from a sample of the sequencing reads of *Saimiri boliviensis* (SRA accession: SRR317821).

Cluster	Read Number	Genome Proportion [%][a]	Proportion of Similarity Hits to Other Clusters[b]	Annotation[c]
1	108845	4.880	3.4000	SINE.Alu
2	68906	3.090	9.3000	SINE.Alu
3[d]	49193	2.210	0.000028	Unclassified
4	23622	1.060	0.4400	LINE.L1/Satellite
5[e]	22193	0.995	0.0012	Satellite.centr
6	15798	0.708	0.1300	LINE.L1
7	11074	0.496	1.1000	LINE.L1/ Satellite
8	9773	0.438	2.5000	LINE.L1/ Satellite
9	8954	0.401	1.5000	LINE.L1
10	7535	0.338	0.0072	LTR.ERVL.MaLR
11	7338	0.329	0.6900	LINE.L1
12	6147	0.276	0.0470	LINE.L1
13	4939	0.221	0.0000	Unclassified
14	4783	0.214	0.0000	Unclassified
15	4723	0.212	0.0000	Unclassified
16	4567	0.205	0.0000	Unclassified
17	2612	0.117	0.0000	DNA.hAT.Charlie
18	2038	0.091	0.0500	LINE.L1
19	1847	0.083	0.0300	LTR.ERVL.MaLR
20	1205	0.054	81.0000	SINE.Alu
21	946	0.042	0.0000	Unclassified
22	861	0.039	0.0000	LTR.ERV1
23	709	0.032	42.0000	SINE.Alu/ SINE.B4
24	656	0.029	0.0260	Unclassified
25	639	0.029	0.5800	LTR.ERV1
26	575	0.026	0.0000	LTR.ERVL
27	497	0.022	1.3000	LINE.L1
28	462	0.021	0.5000	DNA.hAT.Charlie/LTR.ERVL.MaLR
29	454	0.020	0.0000	Unclassified
30	451	0.020	0.0000	Unclassified
31	409	0.018	4.2000	SINE.7SL/srpRNA/SINE.Alu
32	408	0.018	0.6900	LINE.L1/Satellite/LTR.ERVK
33	380	0.017	0.9500	LINE.L1
34	362	0.016	0.0000	LTR.ERVL
35	315	0.014	0.0690	LTR.ERVL.MaLR
36	308	0.014	0.0000	LTR.ERVL.MaLR
37	284	0.013	0.0000	LINE.L1
38	245	0.011	0.0000	LTR.ERVL.MaLR
39	244	0.011	0.0000	LINE.L1
40	232	0.010	0.0000	LTR.ERV1/SINE.MIR/DNA.hAT.Blackjack
41	227	0.010	0.0000	LTR.ERV1

[a] Only clusters with an estimated genome proportion of at least 0.01% of the genome are included.

[b] This proportion indicates whether a significant number of reads from a given cluster also have similarity with reads from other clusters. It is a measure of uniqueness/redundancy of the repeat family.

[c] Annotation is given as displayed in the RepeatExplorer output. Clusters with at least 3% of matching similarity hits with known repeats are annotated accordingly (based on the mammalian repeat library of Repbase). Anonymous clusters are marked as "Unclassified".

[d] This cluster represents the CapA satellite DNA.

[e] This cluster represents the alpha satellite DNA, a well-known component of primate centromeres.
